# SET8 suppression mediates high glucose-induced vascular endothelial inflammation via the upregulation of PTEN

**DOI:** 10.1038/s12276-020-00509-3

**Published:** 2020-10-07

**Authors:** Xuefang Shen, Xiangyuan Chen, Jing Wang, Jing Liu, Zhiyao Wang, Qing Hua, Qichao Wu, Yanguang Su, Huanzhong He, Yuqin Hu, Zhipeng Meng, Wanxia Xiong, Minmin Zhu

**Affiliations:** 1 Department of Anaesthesiology, Fudan University Shanghai Cancer Center, Department of Oncology, Shanghai Medical College, Fudan University, 200032 Shanghai, China; 2 Department of Anaesthesiology, Huzhou Central Hospital, Affiliated Central Hospital of HuZhou University, 313000 Huzhou, Zhejiang China; 3 Department of Anaesthesiology, Huzhou Maternal & Child Health Care Hospital, 313000 Huzhou, Zhejiang China; 4 Department of Anesthesia, Zhongshan Hospital, Fudan University, 200032 Shanghai, China

**Keywords:** Diabetes complications, Vascular diseases

## Abstract

Hyperglycemia-mediated endothelial inflammation participates in the pathogenesis of cardiovascular complications in subjects with diabetes. Previous studies reported that phosphatase and tensin homolog deleted on chromosome ten (PTEN) and SET8 participate in high glucose-mediated endothelial inflammation. In this study, we hypothesize that SET8 regulates PTEN expression, thus contributing to high glucose-mediated vascular endothelial inflammation. Our data indicated that plasma soluble intercellular adhesion molecule-1 (sICAM-1) and endothelial selectin (e-selectin) were increased in patients with diabetes and diabetic rats. PTEN expression was augmented in the peripheral blood mononuclear cells of patients with diabetes and in the aortic tissues of diabetic rats. Our in vitro study indicated that high glucose increased monocyte/endothelial adhesion, endothelial adhesion molecule expression and p65 phosphorylation in human umbilical vein endothelial cells (HUVECs). Moreover, high glucose led to endothelial inflammation via upregulation of PTEN. Furthermore, high glucose inhibited SET8 expression and histone H4 lysine 20 methylation (H4K20me1), a downstream target of SET8. SET8 overexpression reversed the effects of high-glucose treatment. shSET8-mediated endothelial inflammation was counteracted by siPTEN. Furthermore, SET8 was found to interact with FOXO1. siFOXO1 attenuated high glucose-mediated endothelial inflammation. FOXO1 overexpression-mediated endothelial inflammation was counteracted by siPTEN. H4K20me1 and FOXO1 were enriched in the PTEN promoter region. shSET8 increased PTEN promoter activity and augmented the positive effect of FOXO1 overexpression on PTEN promoter activity. Our in vivo study indicated that SET8 was downregulated and FOXO1 was upregulated in the peripheral blood mononuclear cells of patients with diabetes and the aortic tissues of diabetic rats. In conclusion, SET8 interacted with FOXO1 to modulate PTEN expression in vascular endothelial cells, thus contributing to hyperglycemia-mediated endothelial inflammation.

## Diabetes: Protein modulators of vascular inflammation

High glucose levels in patients with diabetes trigger vascular inflammation by affecting the expression of key proteins in blood vessel linings. Elevated glucose causes inflammation of the endothelium, a thin layer of cells that lines blood and lymph vessels, leading to cardiovascular complications. The phosphatase and tensin homolog protein (PTEN) contributes to endothelial inflammation, but the precise mechanisms are unclear. Xuefang Shen at Fudan University in Shanghai, China, and co-workers demonstrated that elevated glucose increases PTEN expression, with increased levels of the protein found in peripheral blood cells of diabetic patients and aortic tissues of diabetic rats. In further experiments on rats, the researchers found that glucose also suppressed another protein called SET8, which contributed to increased PTEN levels. This suggests that SET8 is involved in PTEN modulation, and that both proteins influence vascular inflammation.

## Introduction

The prevalence of diabetes has greatly increased in the last decade, leading to a growing worldwide public health problem^1^. The morbidity and mortality caused by cardiovascular complications are higher in people with diabetes than in people without diabetes^2,3^. It has been indicated that hyperglycemia-mediated endothelial inflammation is involved in the pathogenesis of cardiovascular complications in people with diabetes^4–6^.

Hyperglycemia induces endothelial adhesion molecule expression via the activation of nuclear factor kappa B (NF-κB)^7–9^, thus increasing monocyte/endothelial adhesion^7^ and resulting in endothelial inflammation. The endothelial adhesion molecules that mediate monocyte/endothelial interactions mainly include endothelial selectin (e-selectin) and intercellular adhesion molecule 1 (ICAM-1)^7–9^.

Phosphatase and tensin homolog deleted on chromosome ten (PTEN), located on human chromosome 10q23, functions as a tumor suppressor^10,11^. PTEN has also been reported to contribute to vascular endothelial inflammatory responses^12^. Moreover, PTEN suppression attenuates endothelial NF-κB activity, thus ameliorating vascular endothelial inflammation^13^. As an inducer of endothelial inflammation, PTEN may be an effective target for treating hyperglycemia-induced endothelial inflammation and injury.

SET8, also known as KMT5A, is the only known nucleosome-specific methyltransferase that modifies histone H4 lysine 20 through methylation (H4K20me1)^14^. The methyltransferase activity of SET8 is involved in many important cellular signaling pathways, including DNA repair, cell cycle progression, transcriptional and posttranslational regulation, and cellular metabolism^14,15^. The results from our previous study demonstrated that SET8 suppression contributes to high glucose-induced endothelial adhesion molecule expression in human umbilical vein endothelial cells (HUVECs), thus mediating endothelial inflammation^16^. However, the exact mechanism is still not well understood. In the present study, we hypothesize that SET8 may upregulate PTEN expression in HUVECs, thus playing an important role in high glucose-mediated endothelial inflammation. More importantly, we explored the potential mechanism by which SET8 modulates PTEN expression.

## Materials and methods

### Participants

There were 50 newly diagnosed patients with type 2 diabetes mellitus (T2DM) and 30 healthy volunteers included in the present study. This study conformed to the Declaration of Helsinki and was approved by the Ethics Committee of Huzhou Central Hospital (20191209-01). Written informed consent was acquired from each participant prior to enrollment. The definition of T2DM included the following: fasting plasma glucose levels were ≥126 mg/dl, HbA1c levels were ≥6.5%, plasma glucose levels after 2 h were ≥199.8 mg/dl or a random plasma glucose level was ≥199.8 mg/dl. The exclusion criteria included advanced liver disease, renal failure, valvular heart disease, severe heart failure, stroke, atrial fibrillation, peripheral arterial disease, and other vascular diseases.

### Collection of venous blood samples

In the present study, EDTA vacutainer tubes were used to collect fasting venous blood samples from all participants. The plasma samples were collected and then stored at −80 °C until analysis.

### Collection of peripheral blood mononuclear cells

In the present study, Ficoll standard density gradient centrifugation was employed to collect peripheral blood mononuclear cells. The upper layer containing peripheral blood mononuclear cells was collected and then stored at −80 °C until analysis.

### Rat model of T2DM

Male Sprague-Dawley rats weighing 200–300 g were employed in this study. The rats were acquired from Shanghai SLAC Laboratories. The present study complied with the Guide for the Care and Use of Laboratory Animals of Zhejiang University Laboratory Animal Welfare Ethics Review Committee and was performed according to the Institutional Guidelines for Animal Research and the Guide for the Care and Use of Laboratory Animals published by the US NIH (2011). Ten rats were randomly divided into the control group (con, *n* = 5) and the diabetic group (DM, *n* = 5). For the con group, the rats were injected intraperitoneally once with citrate buffer only (0.1 M, pH 4.5). For the DM group, after feeding the rats a high-sugar/high-fat diet (67% basic feed, 10% lard, 20% sugar, 2.5% cholesterol, and 0.5% sodium cholate) for 2 weeks, the rats received a single intraperitoneal injection of streptozotocin (STZ, 50 mg/kg) and were then moved back to a standard laboratory chow for 4 weeks. Hyperglycemia was verified by detecting blood glucose through tail-neck blood sampling one week after STZ injection.

### Collection of rat blood samples

Rat blood samples were gathered by cardiac puncture. Euthanasia of all the rats was performed by intraperitoneal administration of thiopental sodium (40 mg/kg). Rat blood samples were collected in EDTA vacutainer tubes. The plasma samples were collected and then kept frozen at −80 °C until analysis.

### Detection of sICAM-1 and e-selectin levels

Human and rat sICAM-1 and e-selectin were detected with enzyme-linked immunosorbent assay kits (Meimian Industrial Co., Ltd, Jiangsu, China).

### Cell culture

HUVECs (ATCC; Manassas, USA) were cultured in low-glucose (5 mM) Dulbecco’s modified Eagle’s medium (DMEM; HyClone Laboratories, Logan, USA) with 10% fetal bovine serum and 1% penicillin–streptomycin. The experimental group was cultured in high-glucose (25 mM) DMEM with 10% fetal bovine serum and 1% penicillin–streptomycin for 6 days. THP-1 cells (ATCC; Manassas, USA) were cultured in RPMI 1640 medium (HyClone Laboratories, Logan, USA) with 10% fetal bovine serum and 1% penicillin–streptomycin. The culture environment was created by an incubator containing 5% CO_2_ at 37 °C. The condition of 5 mM glucose plus 20 mM mannitol (Sigma, St. Louis, MO) was employed as an osmotic control in this study.

### Adhesion of mononuclear cells to HUVECs

First, we cultured HUVECs in 5 mM glucose or 25 mM glucose DMEM for 6 days. Then, 30,000 THP-1 cells were added to the HUVECs and coincubated at 37 °C for 30 min. After that, the cells were washed with PBS three times and analyzed by phase-contrast microscopy. We counted five separate culture dishes in 10 microscopic fields of view.

### Western blot analysis

Proteins were extracted from the HUVECs of the different cultures by cell lysis buffer (Cell Signaling Technology, Danvers, MA). Equal amounts of protein were separated by 10% SDS–PAGE gels and then transferred to PVDF membranes (Millipore Corporation). The PVDF membranes were blocked in 5% skim milk at room temperature for 1 h and then incubated with the corresponding primary antibody (1:1000) at room temperature for 2 h. The primary antibodies were monoclonal antibodies against β-actin (ProteinTech, Wuhan, China), PTEN (ProteinTech, Wuhan, China), SET8 (ProteinTech, Wuhan, China), H4K20me1 (Abcam, Cambridge, UK), forkhead box protein O1 (FOXO1) (Cell Signaling Technology, Danvers, MA), and e-selectin (Santa Cruz Biotechnology, Santa Cruz, CA), and polyclonal antibodies against ICAM-1 (Cell Signaling Technology, Danvers, MA) and p-p65 (Cell Signaling Technology, Danvers, MA). The membranes were incubated with the corresponding secondary antibody (1:1000) at room temperature for 1 h. The membranes were then washed, and the proteins were detected by a LAS-4000 mini CCD camera (GE Healthcare).

### RNA extraction and quantitative real-time PCR (qRT-PCR)

Total RNA was obtained by TRIzol (Invitrogen, Grand Island, NY, USA). According to the manufacturer’s instructions, PrimeScript RT reagent (TaKaRa) was used to synthesize complementary DNA (cDNA). Then, we used Hieff UNICON^®^ qPCR TaqMan Probe Master Mix (Yeasen, Shanghai, China) for qPCR on an ABI7500 Real-Time PCR system (Applied Biosystems). The primers used in the present study are shown in Supplementary Table 1.

### Immunohistochemistry (IHC)

The animal tissues were embedded in paraffin and then processed for IHC. The paraffin sections were incubated with anti-SET8 (ProteinTech, Wuhan, China), anti-FOXO1 (Cell Signaling Technology, Danvers, MA), anti-PTEN (Proteintech, Wuhan, China), and anti-CD31 (Proteintech, Wuhan, China) antibodies overnight at 4 °C in a humidified chamber. Double staining was performed with the use of diaminobenzidine and fast red as the enzyme substrates according to the manufacturer’s instructions.

### siRNA, shRNA, and SET8 mutant treatments

HUVECs were transfected with shSET8, a mutant SET8^R295G^ plasmid^15^, siFOXO1 and siPTEN with the use of Lipofectamine 3000 (Invitrogen, USA).

The sequences of shSET8 (Biotend, Shanghai, China) were shRNA-a, 5′-CAACAGAATCGCAAACTTA-3′, and shRNA-b, 5′-CAACAGAATCGCAAACTTA-3′. The sequences of siFOXO-1 (Biotend, Shanghai, China) were siRNA-a, 5′-CCCAGUCUGUCUGAGAUAATT-3′, and siRNA-b, 5′-UUAUCUCAGACAGACUGGGTT-3′. The sequences of siPTEN (Biotend, Shanghai, China) were siRNA-a, 5′-GGUGUAAUGAUAUGUGCAUdTdT-3′, and siRNA-b, 5′-CAAAUUUAAUUGCAGAGUUdTdT-3′.

### Coimmunoprecipitation (Co-IP)

Protein extracts were prepared with the use of cell lysis buffer containing PMSF (Beyotime Biotechnology, Shanghai, China). A total of 30 μl of the cell extract was used as the input. For endogenous IP, the cell extracts were cultured with the corresponding primary antibodies and 50 μl of protein A/G Dynabeads (Thermo Fisher, USA) at 4 °C overnight. Then, 10 μl of the input, IgG, and IP extracts were subjected to western blot.

### Immunofluorescence (IF) staining

HUVECs with the corresponding treatment were seeded onto glass slides. After fixing with 4% paraformaldehyde, the cells were permeabilized with 0.3% Triton X-100 for 5 min and then blocked for 1 h at room temperature. The cells were incubated with anti-SET8 (Proteintech, Wuhan, China) and anti-FOXO1 (Cell Signaling Technology, Danvers, MA) antibodies overnight at 4 °C. 4,6-Diamidino-2-phenylindole (DAPI) was used to stain the nuclei. The images were photographed with a confocal Leica fluorescence microscope.

### Chromatin immunoprecipitation (ChIP) assay

ChIP assays were performed with the use of a Simple ChIP Plus Sonication Chromatin IP Kit (Cell Signaling Technology, MA). Briefly, the cells (1 × 10^7^) were fixed with 1% formaldehyde for 10 min at room temperature to cross-link the DNA and the proteins. The cross-linking reaction was then stopped with the use of glycine. A Microson XL ultrasonic cell disruptor XL (Misonix) was used to shear the chromatin. Ten microliters of the sonicated solution was gathered as an input control. The surplus sonicated solution was incubated with anti-FOXO1 antibody (Cell Signaling Technology, Danvers, MA), anti-H4K20me1 (Abcam, Cambridge, UK) antibody or a negative control IgG at 4 °C overnight. The immunoprecipitants were bound to protein G magnetic beads, and the DNA–protein cross-linking was terminated by incubation at 65 °C for 2 h. After purification, the enriched DNA sequences were detected by PCR. To confirm whether FOXO1 binds to the methylated promoter of PTEN, a re-ChIP assay was performed. In brief, after the standard ChIP assay, the beads were incubated with 10 mM dithiothreitol for 30 min at 37 °C. The eluent was then diluted with sonication buffer, followed by a second round of the ChIP assay. The PTEN oligonucleotide primer sequences were forward, 5′-TTGGATGTGGGTGCTTGTGT-3′, and reverse, 5′-CTTCTTCCTTTGCTCGGGGT-3′.

### Dual-luciferase assay

The effect of SET8 and FOXO1 on the activity of the PTEN promoter was evaluated by a Promega Dual-luciferase Assay Kit (Madison, WI, USA). The PTEN promoter was amplified and ligated into the pGL3-basic vector to create the pGL3–PTEN construct. The pGL3–PTEN plasmid was transfected along with a Renilla luciferase vector into HUVECs. The effect of SET8 and FOXO1 on PTEN promoter activity was assessed using a dual-luciferase assay kit.

### Statistical analysis

The data were acquired from at least five experiments performed separately, and the results are shown as the mean ± SD (standard deviation). Two-tailed unpaired *t*-tests or one-way ANOVAs performed with GraphPad Prism Version 7.0 (GraphPad Software, San Diego, CA) were used to compare the groups. *P* < 0.05 was considered significant.

## Results

### e-selectin, sICAM-1, and PTEN expression levels were increased in patients with diabetes and rats

**Fig. 1:**
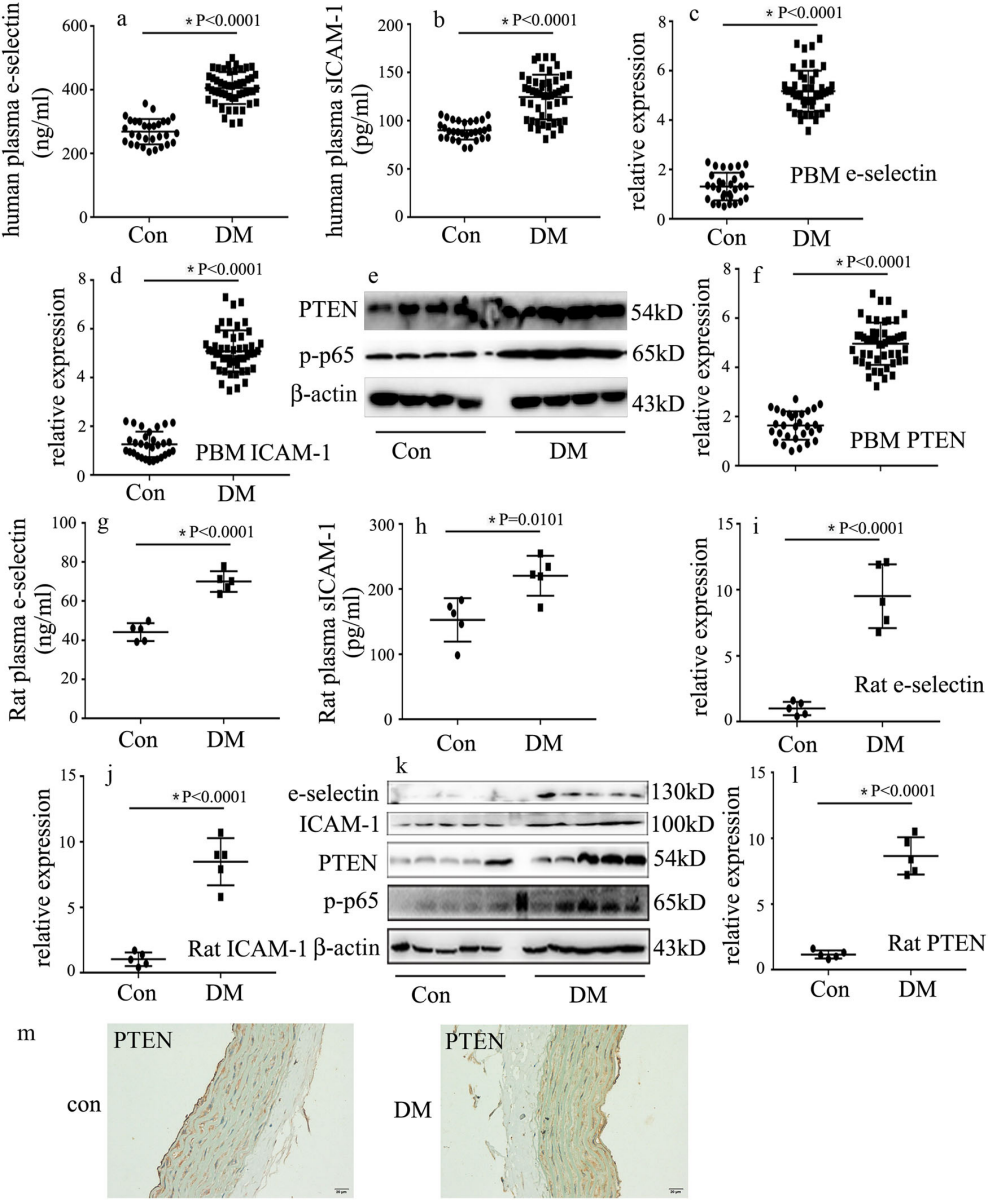
e-selectin, sICAM-1, and PTEN expression levels were increased in patients with diabetes and rats. **a** Plasma e-selectin was measured in patients with diabetes and healthy volunteers (con: *n* = 30, DM: *n* = 50). **b** Plasma sICAM-1 was measured in patients with diabetes and healthy volunteers (con: *n* = 30, DM: *n* = 50). **c** The mRNA expression of e-selectin was examined by qPCR in peripheral blood mononuclear cells in the subjects (con: *n* = 30, DM: *n* = 50). **d** The mRNA expression of sICAM-1 was examined by qPCR in peripheral blood mononuclear cells in the subjects (con: *n* = 30, DM: *n* = 50). **e** Results from the western blot analysis of PTEN and p-p65 expressions in peripheral blood mononuclear cells in the subjects (con: *n* = 30, DM: *n* = 50). **f** The mRNA expression of PTEN was examined by qPCR in peripheral blood mononuclear cells in the subjects (con: *n* = 30, DM: *n* = 50). **g** Plasma e-selectin was measured in the control group and diabetic group rats (*n* = 5/group). **h** Plasma sICAM-1 was measured in the control group and diabetic group rats (*n* = 5/group). **i** The mRNA expression of e-selectin was examined by qPCR in rat aortic tissues in the control group and the diabetic group (*n* = 5/group). **j** The mRNA expression of ICAM-1 was examined by qPCR in rat aortic tissues from the control group and the diabetic group (*n* = 5/group). **k** Results from the western blot analysis of e-selectin, ICAM-1, PTEN, and p-p65 expression in rat aortic tissues from the control group and the diabetic group (*n* = 5/group). **l** The mRNA expression of PTEN was examined by qPCR in rat aortic tissues from the control group and the diabetic group (*n* = 5/group). **m** Immunostaining of PTEN in rat aortic tissues from the control group and the diabetic group (*n* = 5/group). Scale bar, 20 μm. PBM peripheral blood mononuclear cells. *P < 0.05, compared with the control group.

Endothelial adhesion molecule overexpression leads to endothelial inflammation, thus mediating vascular endothelial injury in hyperglycemia^4–6^. The characteristics of the subjects are shown in Table 1. The levels of fasting blood sugar (FBS) and glycated hemoglobin (HbA1c) in the patients with T2DM were higher than those in the healthy volunteers. Moreover, plasma e-selectin (Fig. 1a) and sICAM-1 (Fig. 1b) were augmented in patients with T2DM. Previous studies have shown that NF-κB activation participates in ICAM-1 and e-selectin overexpressions^7–9^, and that PTEN activates NF-κB^13^. Therefore, we sought to determine p-p65 and PTEN expressions in patients with T2DM. One previous study detected the specific protein expression of peripheral blood mononuclear cells to reflect the protein levels in the blood vessels of patients with diabetes^17^. Thus, in this study, e-selectin, ICAM-1, p-p65, and PTEN expression levels in peripheral blood mononuclear cells in subjects were measured. Compared with the healthy volunteers, the protein and/or mRNA levels of e-selectin, ICAM-1, p-p65, and PTEN were increased in the peripheral blood mononuclear cells of patients with T2DM (Fig. 1c-f).

**Table 1:** The baseline characteristics of the subjects.

Variables	Con	DM	*P*-value
Male/female	13/17	28/22	0.76
Age (years)	53.1 ± 12.9	58.3 ± 13.6	0.09
BMI (kg/m^2^)	23.2 ± 2.6	24.9 ± 4.2	0.41
SBP (mmHg)	125.4 ± 13.3	126.7 ± 15.2	0.69
DBP (mmHg)	69.7 ± 10.7	68.3 ± 9.7	0.53
FBS (mg/dl)	90.0 ± 10.8	253.8 ± 102.6	<0.0001
HbA1C (%)	5.6 ± 0.8	10.4 ± 2.5	<0.0001

*BMI* body mass index, *SBP* systolic blood pressure, *DBP* diastolic bllod pressure, *FBS* fasting blood sugar, *HbA1c* glycated hemoglobin.

Blood glucose concentrations of diabetic rats were significantly higher than those of the control group (Supplementary Fig. 1). Consistently, plasma levels of e-selectin (Fig. 1g) and sICAM-1 (Fig. 1h) in diabetic rats, in addition to the protein and/or mRNA levels of e-selectin, ICAM-1, p-p65, and PTEN in the aortic tissues of diabetic rats, were higher than those of the control group (Fig. 1i–m).

### High glucose levels mediated endothelial inflammation via the upregulation of PTEN expression in HUVECs

**Fig. 2:**
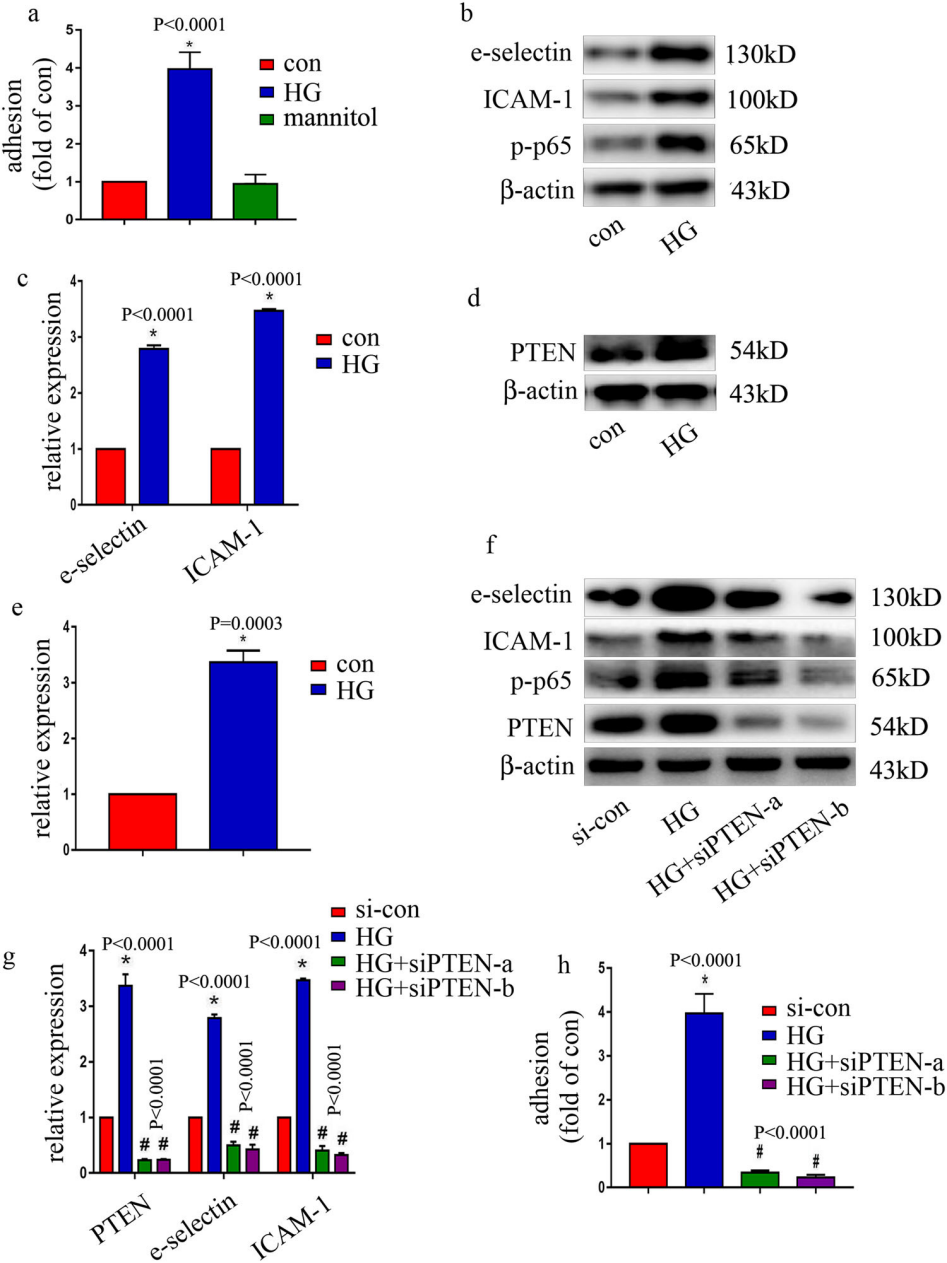
High glucose mediated endothelial inflammation via the upregulation of PTEN expression in HUVECs. **a** Monocyte/endothelial adhesion under normal, high-glucose or osmotic control conditions was measured. **b** Results from the western blot analysis of e-selectin, ICAM-1, and p-p65 expression in HUVECs cultured under normal or high-glucose conditions. **c** mRNA expression of e-selectin and ICAM-1 was examined by qPCR in cells grown in normal or high-glucose medium. **d** Results from the western blot analysis of PTEN expression in HUVECs cultured under normal or high-glucose conditions. **e** mRNA expression of PTEN was measured by qPCR in cells grown in normal or high-glucose medium. **f** The effects of siPTEN on high glucose-induced e-selectin and ICAM-1 expressions, and p65 phosphorylation in HUVECs were assessed by western blot analysis. **g** The effects of siPTEN on high glucose-induced e-selectin and ICAM-1 expressions in HUVECs were assessed by qPCR analysis. **h** The effects of siPTEN on high glucose-induced monocyte/endothelial adhesion were measured. *P < 0.05, compared with the control group; ^#^*P* < 0.05, compared with the high-glucose treatment group, *n* = 5/group.

To explore the potential mechanism by which ICAM-1, e-selectin, and PTEN were increased in patients with T2DM and diabetic rats, HUVECs were employed. To determine whether high glucose can induce endothelial inflammation, HUVECs were incubated in different types of medium containing either a normal glucose level (con, 5 mM, 6 days) or a high glucose level (HG, 25 mM, 6 days). The results indicated that high glucose increased monocyte/endothelial adhesion (Fig. 2a) and increased e-selectin and ICAM-1 protein levels (Fig. 2b) and mRNA expression (Fig. 2c) in HUVECs. Mannitol had no effect on monocyte/endothelial adhesion (Fig. 2a). Moreover, high glucose induced p65 phosphorylation (Fig. 2b). Previous studies have shown that PTEN activates NF-κB^13^; therefore, we sought to determine PTEN expression in HUVECs. We found that the high-glucose treatment increased PTEN expression (Fig. 2d, e). To further clarify whether PTEN is involved in p-p65 and endothelial adhesion molecule overexpression in hyperglycemic HUVECs, two independent siRNAs against PTEN were employed. The effects of siPTEN were validated by the western blot (Fig. 2f) and qRT-PCR results (Fig. 2g). Our data showed that siPTEN decreased high glucose-induced e-selectin and ICAM-1 expressions (Fig. 2f, g), inhibited high glucose-induced p65 phosphorylation (Fig. 2f) and reduced monocyte/endothelial interactions (Fig. 2g) in hyperglycemic HUVECs. These data indicated that PTEN positively regulated p65 phosphorylation, thus inducing endothelial adhesion molecule expression and monocyte/endothelial interaction in hyperglycemic HUVECs.

### SET8 downregulation mediated high glucose-induced endothelial inflammation by increasing PTEN expression in HUVECs

**Fig. 3:**
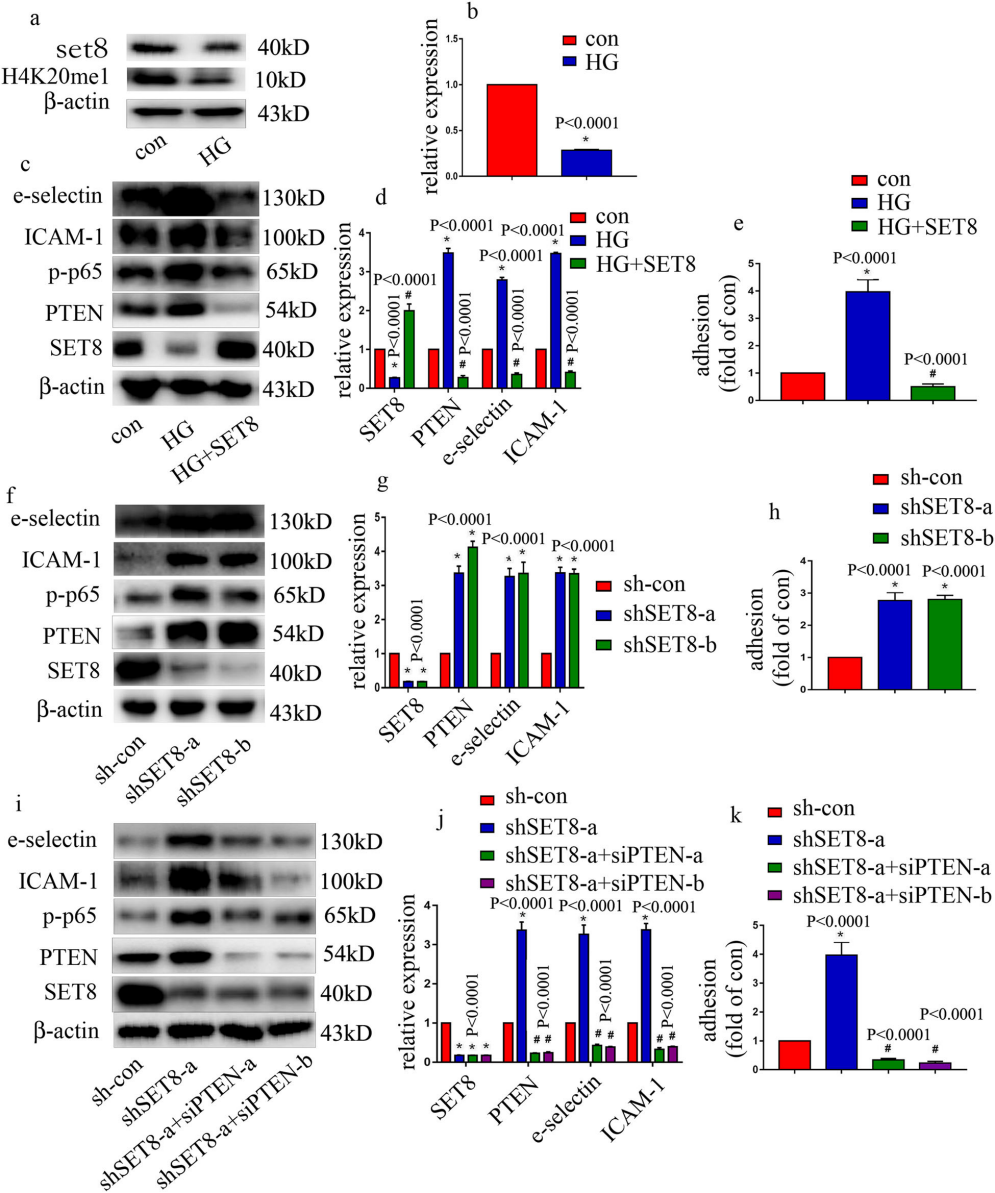
SET8 overexpression reversed high glucose-mediated endothelial inflammation. **a** Results from the western blot analysis of SET8 and H4K20me1 in HUVECs, HUVEC nuclei, and HUVEC cytoplasm cultured under normal or high-glucose conditions. **b** qPCR analysis of SET8 in HUVECs cultured under normal or high-glucose conditions. **c** Results from the western blot analysis of SET8, PTEN, p-p65, e-selectin, and ICAM-1 expressions in HUVECs overexpressing SET8 under high-glucose conditions. **d** qPCR analysis of SET8, PTEN, p-p65, e-selectin, and ICAM-1 expressions in HUVECs overexpressing SET8 under high-glucose conditions. **e** Monocyte/endothelial adhesion under normal or high-glucose conditions and with SET8-overexpressing HUVECs under high-glucose conditions was measured. **f** Results from the western blot analysis of SET8, PTEN, p-p65, e-selectin, and ICAM-1 expressions in HUVECs expressing shSET8. **g** Results from the qPCR analysis of SET8, PTEN, p-p65, e-selectin, and ICAM-1 expressions in HUVECs expressing shSET8. **h** Monocyte/endothelial adhesion under normal conditions and in the presence of HUVECs expressing shSET8 was measured. **i** The results from the western blot analysis of SET8, PTEN, p-p65, e-selectin, and ICAM-1 expressions in HUVECs in which SET8 was silenced and PTEN was downregulated. **j** Results from the qPCR analysis of SET8, PTEN, e-selectin, and ICAM-1 expressions in HUVECs in which SET8 was silenced and PTEN was downregulated. **k** Monocyte/endothelial adhesion in the presence of HUVECs expressing shSET8 or shSET8+siPTEN was measured. *P < 0.05, compared with the control group; ^#^*P* < 0.05, compared with the high-glucose treatment or shSET8 treatment group, *n* = 5/group.

The results from our previous study indicated that SET8 suppression in HUVECs mediates high glucose-induced endothelial inflammation^14^. Consistently, SET8 was found to be downregulated by high-glucose treatment in the present study (Fig. 3a, b). The levels of H4K20me1, a downstream target of SET8, were also significantly decreased by high-glucose treatment (Fig. 3a). To investigate the effect of SET8 on high glucose-mediated HUVEC inflammation, both loss-of-function and gain-of-function approaches were employed. Our data indicated that SET8 overexpression counteracted high glucose-induced PTEN, p-p65, and endothelial adhesion molecule expression in HUVECs (Fig. 3c, d) and reversed the high glucose-induced monocyte/endothelial interactions (Fig. 3e). Moreover, the effects of shSET8 were similar to those of the high-glucose treatment (Fig. 3f-h). To explore whether the effects of shSET8 were achieved via the upregulation of PTEN expression, we knocked down PTEN in HUVECs in which SET8 was downregulated. The results showed that PTEN downregulation reversed SET8 silencing-induced p-p65 and endothelial adhesion molecule expression (Fig. 3i, j), and counteracted shSET8-induced monocyte/endothelial adhesion (Fig. 3k). These data indicated that SET8 downregulation mediated endothelial inflammation via the upregulation of PTEN expression.

### SET8 interacted with FOXO1

**Fig. 4:**
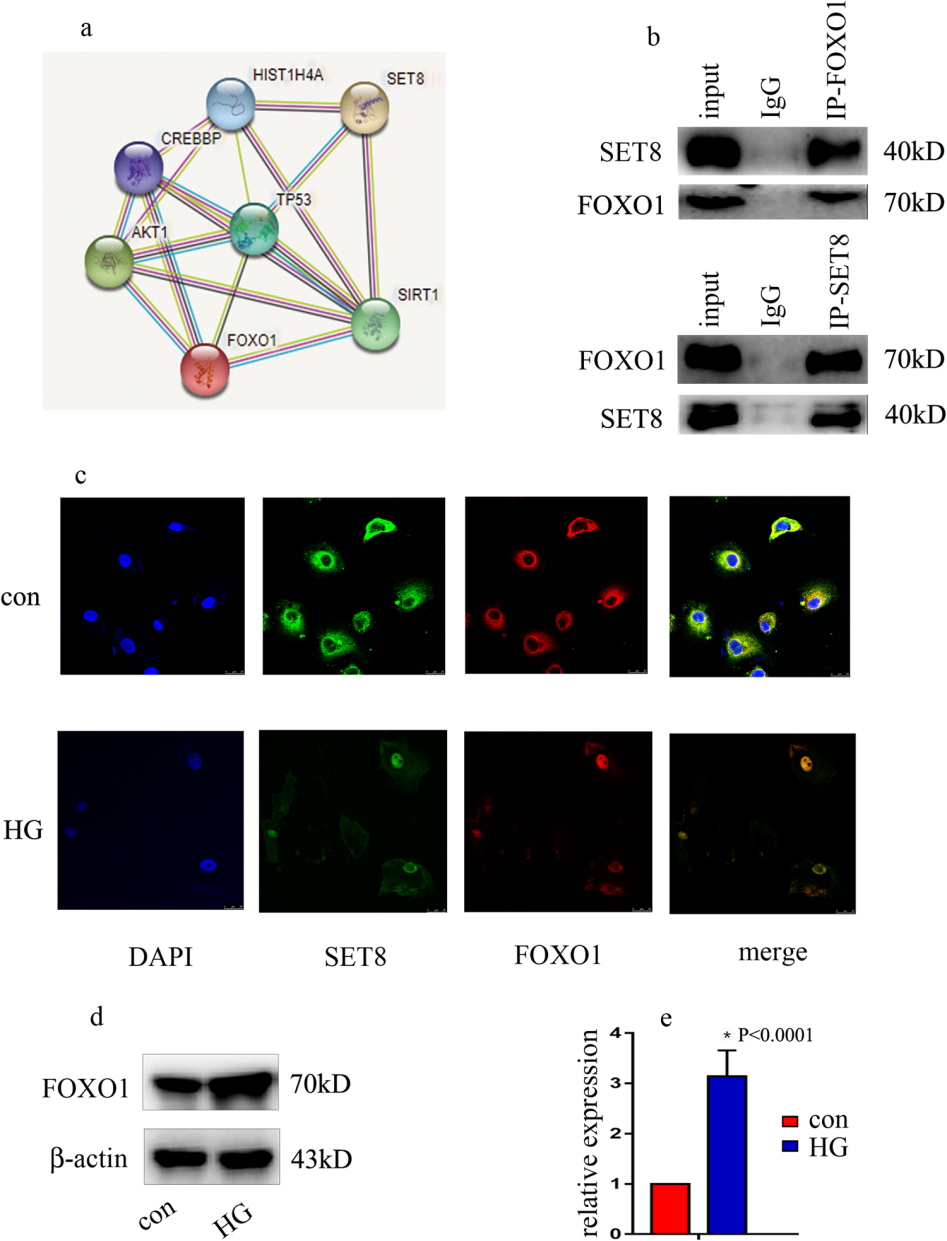
SET8 interacted with FOXO1. **a** Several proteins that interact with SET8 are shown in Fig. 3a (https://string-db.org). **b** Interactions between SET8 and FOXO1 in HUVECs were verified by Co-IP. **c** Colocalization of SET8 and FOXO1 in HUVECs was determined by confocal microscopy. **d** Results of the western blot analysis of FOXO1 in HUVECs cultured under normal or high-glucose conditions. **e** Results from the qPCR analysis of FOXO1 in HUVECs cultured under normal or high-glucose conditions. *P < 0.05, compared with the control group, *n* = 5/group.

To discover the potential regulatory mechanism using bioinformatics, we predicted the proteins that interacted with SET8. Several transcription factors that interact with SET8 are shown in Fig. 4a (https://string-db.org). Co-IP experiments verified the interaction between SET8 and FOXO1 in HUVECs (Fig. 4b). Double immunofluorescent staining revealed the colocalization of SET8 and FOXO1 in the nucleus (Fig. 4c). Moreover, our data indicated that high-glucose treatment induced SET8 and FOXO1 nuclear translocation in in vivo studies (Fig. 4c). These data showed that high glucose regulated the cellular distribution of SET8 (Fig. 4c), as well as its gene expression (Fig. 3a), in HUVECs. Furthermore, our data indicated that FOXO1 was upregulated by high-glucose treatment (Fig. 4d, e).

### FOXO1 overexpression was involved in high glucose-mediated inflammation via the upregulation of PTEN expression in HUVECs

**Fig. 5:**
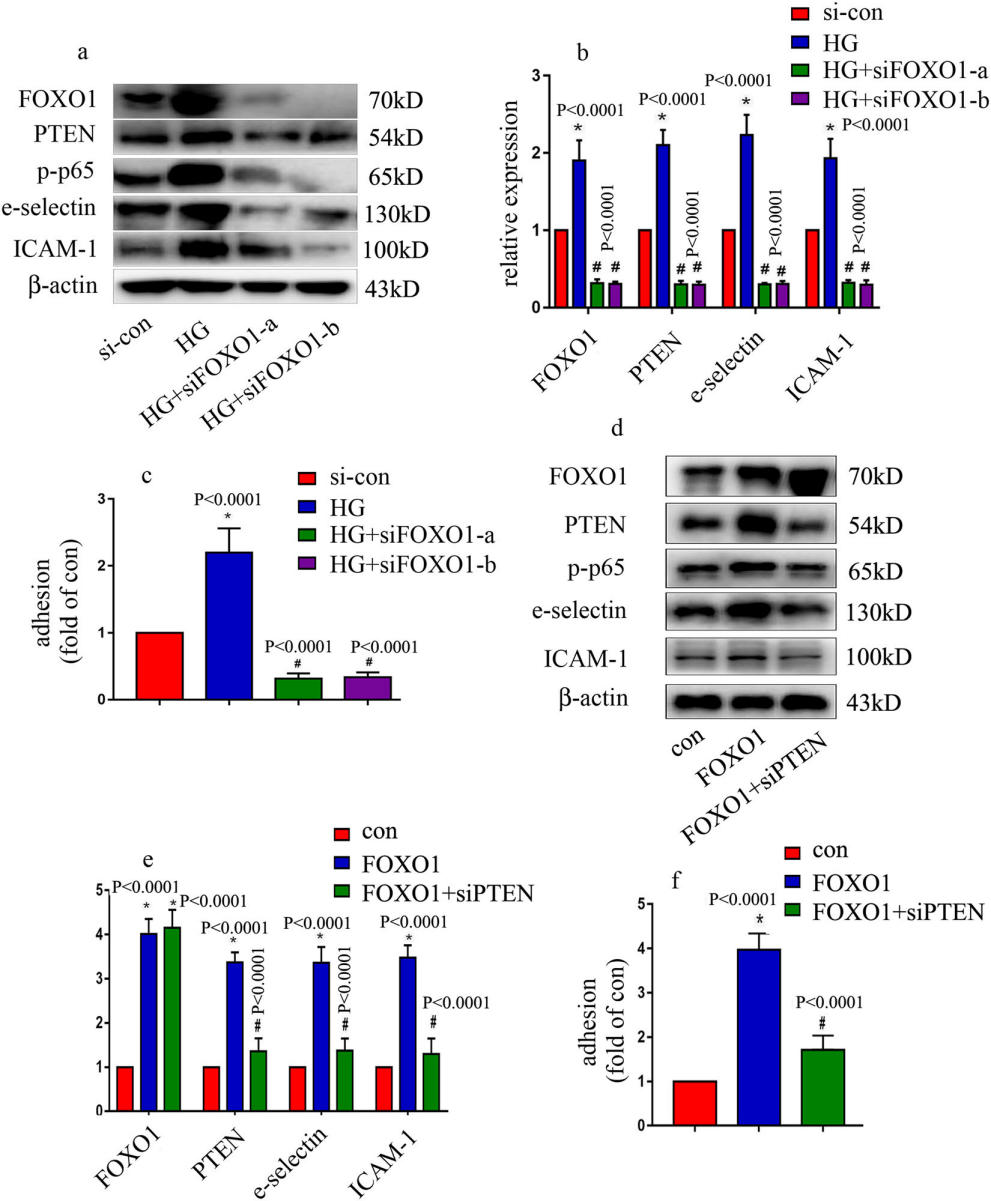
FOXO1 downregulation reversed high glucose-mediated endothelial inflammation. **a** Results from the western blot analysis of PTEN, p-p65, e-selectin, and ICAM-1 expressions in HUVECs expressing siFOXO1 under high-glucose conditions. **b** Results from the qPCR analysis of PTEN, p-p65, e-selectin, and ICAM-1 expressions in HUVECs expressing siFOXO1 under high-glucose conditions. **c** The effects of siFOXO1 on high glucose-induced monocyte/endothelial adhesion were measured. **d** Results from the western blot analysis of PTEN, p-p65, e-selectin, and ICAM-1 expressions in HUVECs with siPTEN under FOXO1 overexpression conditions. **e** Results from the qPCR analysis of PTEN, p-p65, e-selectin, and ICAM-1 expressions in HUVECs with siPTEN and overexpressing FOXO1. **f** The effects of siPTEN on FOXO1 overexpression-induced monocyte/endothelial adhesion were measured. *P < 0.05, compared with the control group; ^#^*P* < 0.05, compared with the high-glucose treatment group, *n* = 5/group.

To investigate the effect of FOXO1 on high glucose-mediated HUVEC inflammation, both loss-of-function and gain-of-function approaches were used. siFOXO1 counteracted the high glucose-induced p65 phosphorylation and endothelial adhesion molecule expression (Fig. 5a, b) and reversed the high glucose-induced monocyte/endothelial interaction (Fig. 5c). Moreover, the effects of FOXO1 overexpression were similar to those of the high-glucose treatment (Fig. 5d–f). To explore whether the effects of FOXO1 overexpression were achieved via the upregulation of PTEN expression, we knocked down PTEN in FOXO1-overexpressing HUVECs. The results showed that PTEN downregulation reversed FOXO1 overexpression-induced phosphorylation of p65 and endothelial adhesion molecule expression (Fig. 5d, e), and counteracted FOXO1 overexpression-induced monocyte/endothelial adhesion (Fig. 5f). These data indicated that FOXO1 upregulation increased PTEN expression in hyperglycemic HUVECs, thus contributing to high glucose-mediated endothelial inflammation.

### SET8 interacted with FOXO1 to modulate PTEN transcriptional activity in HUVECs

**Fig. 6:**
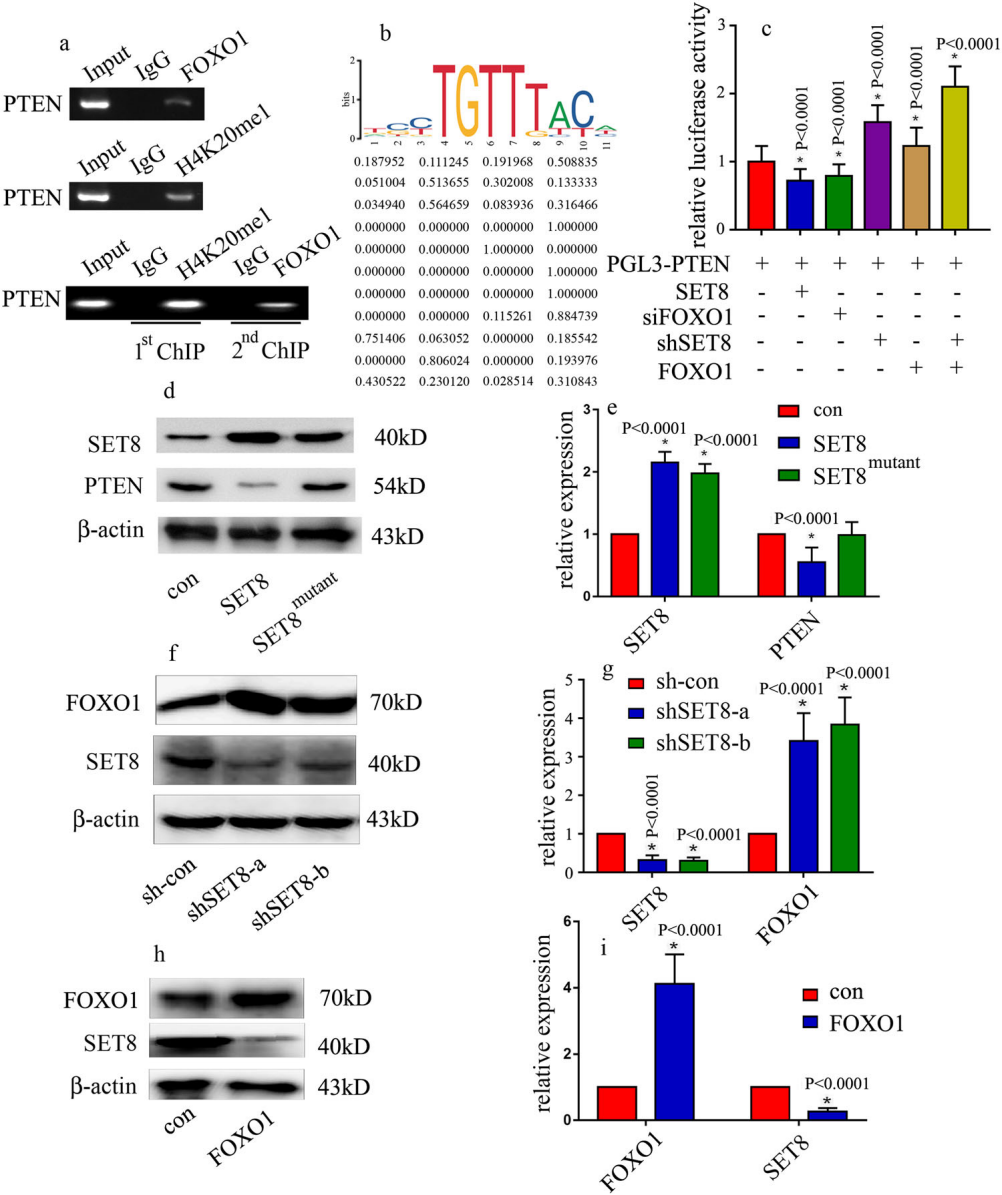
SET8 interacted with FOXO1 to modulate PTEN expression in HUVECs. **a** H4K20me1 and FOXO1 were enriched in the PTEN promoter region. The re-ChIP assay results indicated that H4K20me1 and FOXO1 occupied the same region of the PTEN promoter. **b** The putative FOXO1-binding site of PTEN. The motif logo and position weight matrix are shown in the upper and lower panels, respectively. **c** PTEN promoter activity was detected by luciferase reporter assays. *P < 0.05, compared with the control group. **d** Results from the western blot analysis of SET8 and PTEN expressions in HUVECs compared with HUVECs serving as the control or those overexpressing SET8 or mutant SET8^R259G^. **e** Results from the qPCR analysis of SET8 and PTEN expressions in HUVECs compared with HUVECs serving as the control or those overexpressing SET8 or mutant SET8^R259G^. **f** Results from the western blot analysis of SET8 and PTEN expressions in HUVECs compared with HUVECs serving as the control or those transfected with shSET8. **g** Results from the qPCR analysis of SET8 and PTEN expressions in HUVECs compared with HUVECs serving as the control or those transfected with shSET8. **h** Results from the western blot analysis of SET8 and PTEN expressions in HUVECs compared with HUVECs serving as the control or those overexpressing FOXO1. **i** Results from the qPCR analysis of SET8 and PTEN expressions in HUVECs compared with HUVECs serving as the control or those overexpressing FOXO1. *P < 0.05, compared with the control group, *n* = 5/group.

Next, to determine whether PTEN is targeted by SET8 and FOXO1, we examined the genome-wide distribution of H4K20me1 and FOXO1 in HUVECs by a ChIP assay. Our data indicated that both H4K20me1 and FOXO1 were enriched in the PTEN promoter region (Fig. 6a). To confirm whether FOXO1 binds to the methylated promoter of PTEN, we performed a sequential re-ChIP assay and observed that H4K20me1 and FOXO1 occupied the same region of the PTEN promoter (Fig. 6a). The data indicated that FOXO1 bound to the H4K20me1 methylated promoter of PTEN. The putative FOXO1-binding site is shown in Fig. 6b. The motif logo and position weight matrix are shown in the upper and lower panels, respectively (Fig. 6b). Moreover, luciferase reporter assays indicated that SET8 overexpression and siFOXO1 both reduced PTEN promoter activity (Fig. 6c). shSET8 not only increased PTEN promoter activity but also increased the positive effect of FOXO1 overexpression on PTEN promoter activity (Fig. 6c). These data demonstrated that SET8 interacted with FOXO1 to regulate PTEN promoter activity in hyperglycemic HUVECs. Furthermore, SET8 overexpression attenuated PTEN expression, while mutant SET8 ^R259G^ did not affect PTEN expression (Fig. 6d, e). These data demonstrated that SET8-mediated H4K20me1 was necessary to regulate PTEN expression in HUVECs. Furthermore, shSET8 increased FOXO1 expression in HUVECs (Fig. 6f, g). Consistently, FOXO1 overexpression inhibited SET8 expression (Fig. 6h, i). These data indicated that SET8 and FOXO1 inhibited each other in HUVECs.

### The SET8 decrease and FOXO1 increase were confirmed in patients with diabetes and rats

**Fig. 7:**
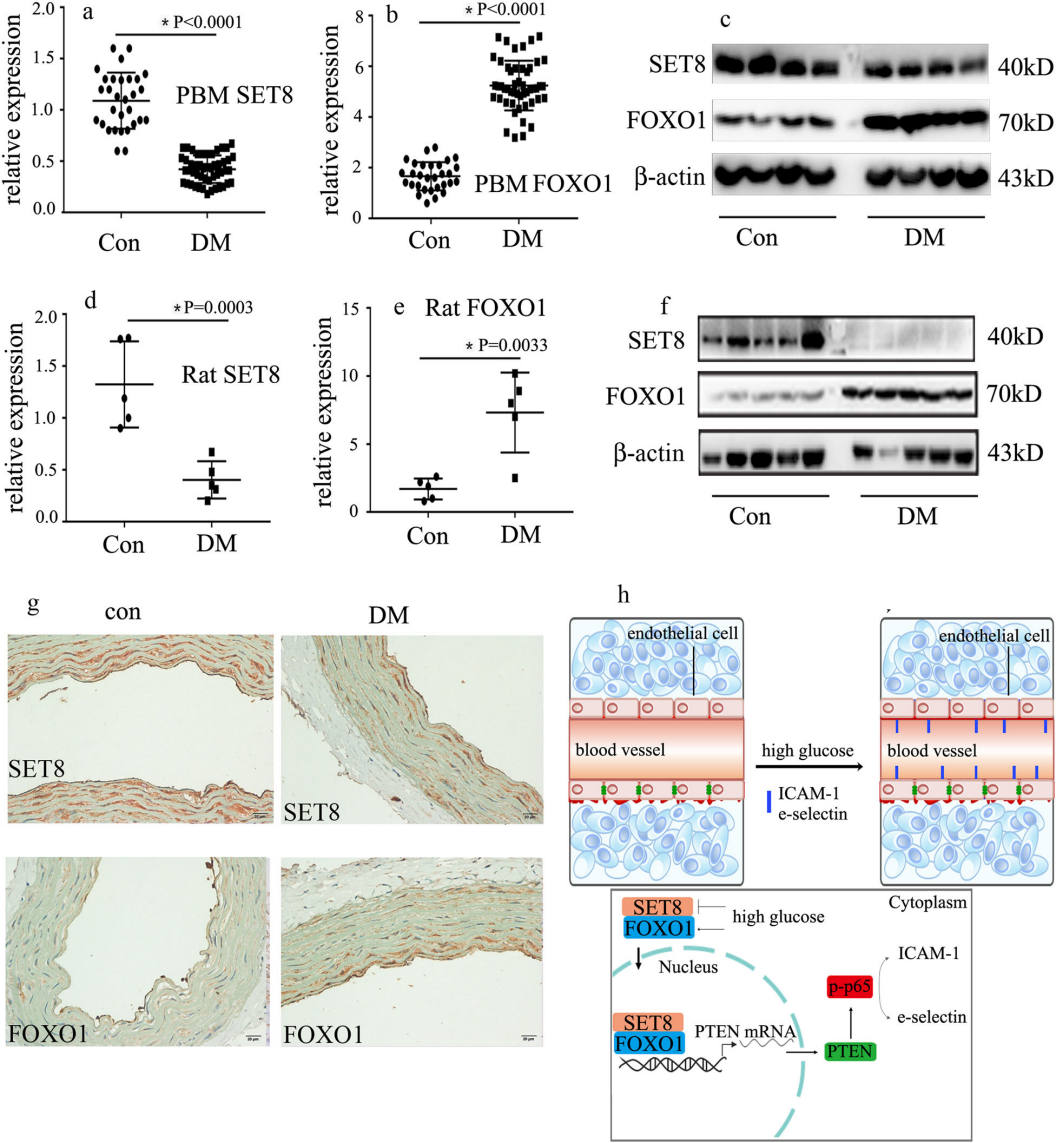
The SET8 decrease and FOXO1 increase were confirmed in patients with diabetes and rats. **a** The mRNA expression of SET8 was examined by qPCR in the peripheral blood mononuclear cells of patients with diabetes and healthy volunteers (con: *n* = 30, DM: *n* = 50). **b** The mRNA expression of FOXO1 was examined by qPCR in peripheral blood mononuclear cells from patients with diabetes and healthy volunteers (con: *n* = 30, DM: *n* = 50). **c** Results from the western blot analysis of SET8 and FOXO1 expressions in peripheral blood mononuclear cells in patients with diabetes and healthy volunteers (con: *n* = 30, DM: *n* = 50). **d** The mRNA expression of SET8 was examined by qPCR in rat aortic tissues from the control group and the diabetic group (*n* = 5/group). **e** The mRNA expression of FOXO1 was examined by qPCR in rat aortic tissues from the control group and the diabetic group (*n* = 5/group). **f** Results from the western blot analysis of SET8 and PTEN expressions in rat aortic tissues from the control group and the diabetic group (*n* = 5/group). **g** Immunostaining of SET8 and FOXO1 in rat aortic tissues from the control group and the diabetic group (*n* = 5/group). Scale bar, 20 μm. **h** Schematic representation of the working model. PBM peripheral blood mononuclear cells. *P < 0.05, compared with the control group.

To determine whether the protein and mRNA levels of SET8 and FOXO1 in patients with diabetes and rats were consistent with those of hyperglycemic HUVECs, we assessed SET8 and FOXO1 expressions in the peripheral blood mononuclear cells of patients with diabetes and the aortic tissues of diabetic rats. Our data show that SET8 decreased and FOXO1 increased in the peripheral blood mononuclear cells of patients with diabetes (Fig. 7a–c) and the aortic tissues of diabetic rats (Fig. 7d–g). In conclusion, the present study indicated that FOXO1 and SET8 interacted to modulate PTEN expression, thus mediating endothelial inflammation in hyperglycemic HUVECs (Fig. 7h).

## Discussion

The main finding of the present study suggests that high glucose, via the upregulation of PTEN expression, induced p65 phosphorylation and adhesion molecule expression, thus mediating endothelial cell inflammation. Moreover, high-glucose treatment attenuated SET8 expression while increasing FOXO1 expression. Furthermore, H4K20me1 and FOXO1 were enriched in the PTEN promoter region. Mechanistic studies demonstrated that SET8 interacted with the transcription factor FOXO1 to modulate PTEN transcriptional activity in hyperglycemic HUVECs, thus mediating endothelial inflammation.

Hyperglycemia plays a vital role in vascular inflammation^18^, which contributes to the pathogenesis of cardiovascular complications in people with diabetes^4–6^. PTEN overexpression mediates endothelial inflammatory responses in coronary artery endothelial cells via inhibition of the phosphatidylinositol-3-kinase/protein kinase B signaling pathway^12^. Moreover, PTEN was found to participate in endothelial inflammation via NF-κB activation in HUVECs^13^. Similarly, PTEN suppression was found to attenuate myocardial ischemic injury^19^ and cardiac dysfunction after myocardial infarction^20^. These data indicated that PTEN may be an effective target for treating cardiovascular inflammation and injury. Indeed, in the present study, we found that high glucose increased PTEN expression (Fig. 2d, e), augmented p65 phosphorylation (Fig. 2b), induced adhesion molecule expression (Fig. 2b, c) and increased monocyte/endothelial interactions (Fig. 2a). Moreover, siPTEN in HUVECs reversed high glucose-mediated endothelial inflammation (Fig. 2f–h). These data indicate that high glucose-mediated endothelial inflammation via the upregulation of PTEN expression.

In a previous study, we found that SET8 downregulation was involved in high glucose-induced adhesion molecule expression in HUVECs, thus mediating endothelial inflammation^14^. Moreover, SET8 suppression induced the expression of proinflammatory enzymes^21^ and mediated NOD-like receptor pyrin domain 3 inflammasome activation in hyperglycemic HUVECs^22^. In this study, SET8 overexpression was found to inhibit high glucose-induced PTEN, p-p65, and adhesion molecule expression, thus alleviating high glucose-mediated endothelial inflammation (Fig. 3c, d). Moreover, H4K20me1, a downstream target of SET8, was enriched in the PTEN promoter region (Fig. 6a). Furthermore, siPTEN reversed SET8 knockdown-induced p-p65 and endothelial adhesion molecule expression (Fig. 3i–k). These data indicated that SET8 overexpression inhibited high glucose-induced p-p65 and adhesion molecule expression via the suppression of PTEN.

FOXO1 is a member of the forkhead family of transcription factors. FOXO1 plays an essential role in cell death, cellular metabolism, and oxidation resistance in vascular endothelial cells^23,24^. Recently, FOXO1 was found to be essential for vessel-related complications in patients with diabetes^25^. Moreover, FOXO1 knockout ameliorated cardiomyocyte dysfunction and insulin resistance in diabetic mice^26^. Endothelial FOXO1 deletion has been shown to prevent atherosclerosis and elevate the number of skeletal muscle capillaries in studies in vivo^27,28^. FOXO1 inhibition attenuated pericyte apoptosis in diabetic retinopathy^29^. The findings from these studies indicate that FOXO1 plays an essential role in the process of diabetes. In the present study, we found that high glucose induced FOXO1 expression in HUVECs (Fig. 4d, e) and endothelial inflammation (Fig. 2a–c). Moreover, siFOXO1 inhibited high glucose-induced PTEN expression and endothelial inflammation (Fig. 5a–c). Furthermore, FOXO1 overexpression induced endothelial inflammation, which could be reversed by siPTEN (Fig. 5d–f). These data indicated that FOXO1 in hyperglycemic HUVECs was involved in high glucose-mediated endothelial inflammation via the upregulation of PTEN expression.

Studies have demonstrated that the transcriptional activity of FOXO1 could be regulated by epigenetic changes in hyperglycemic vascular endothelial cells^30,31^. In the present study, we found an interaction between the SET8 and FOXO1 proteins (Fig. 4b, c). Moreover, FOXO1 (Fig. 6a) and H4K20me1 FOXO1 (Fig. 6b) both occupy the PTEN promoter region. Furthermore, shSET8 augmented the positive effect of FOXO1 overexpression on PTEN promoter activity (Fig. 6c). These data indicated that SET8 interacted with FOXO1 to modulate PTEN expression in hyperglycemic HUVECs, thus mediating endothelial inflammation. Furthermore, SET8 overexpression attenuated PTEN expression, while mutant SET8^R259G^ did not affect PTEN expression (Fig. 5d, e). These data indicated that SET8-mediated H4K20me1 was involved in the modulation of PTEN expression.

The study has some limitations. First, whether SET8 interacts with FOXO1 directly or indirectly deserves further research. However, our data may indicate that the SET8/FOXO1 interaction counteracted the positive effect of FOXO1 on PTEN promoter activity. Moreover, our data demonstrated that SET8-mediated H4K20me1 was necessary to regulate the transcriptional activity of FOXO1 in HUVECs. Second, this study was performed using HUVECs, and our findings need to be validated using other primary endothelial cell models. Third, mechanistic studies were mainly carried out in HUVECs, and the results should be confirmed in an in vivo study. Fourth, the mechanism by which SET8 and FOXO1 inhibit each other deserves further research. Fifth, how PTEN enhances the phosphorylation of p65 in hyperglycemic HUVECs deserves further research.

In conclusion, we observed that SET8 expression was decreased, FOXO1 and PTEN expression was increased, and endothelial inflammation was augmented in people with diabetes and diabetic rats. The present study also indicated that high glucose increased PTEN expression in hyperglycemic HUVECs, thus mediating endothelial inflammation. Moreover, high glucose attenuated SET8 expression and augmented FOXO1 expression. Furthermore, SET8 interacted with FOXO1 to regulate PTEN expression in hyperglycemic HUVECs, which contributed to high glucose-mediated endothelial inflammation.

## Supplementary information

supplementary figures, supplementary tables, and supplementary figure legends
