# Supplementary material for: SET8 suppression mediates high glucose-induced vascular endothelial inflammation via the upregulation of PTEN
Source: Exp Mol Med. 2020 Oct 7;52(10):1715–29. doi: 10.1038/s12276-020-00509-3 (PMC8080680; doi:10.1038/s12276-020-00509-3)
Supplement: Supplementary file 1 — supplementary figures, supplementary tables, and supplementary figure legends [file 12276_2020_509_MOESM1_ESM.pdf]

Supplementary table 1 Primers used for real-time RT-PCR analysis.

species		RNA sequence
Human		
	Set8	F 5'-ACGGCGTACCTCACCTCATCC-3'
		R 5'-TGCGGTCCCCATAGTCATACAGG-3'
	Foxo1	F 5'-CTGGAGGAGAGCGAGGACTTCC-3'
		R 5'-TGGTGATGAGGTCGGCGTAGG-3'
	PTEN	F 5'-TTCCCAGTCAGAGGCGCTAT-3'
		R 5'-ACAGGTAACGGCTGAGGGAA-3'
	ICAM-1	F 5'-TGCAAGAAGATAGCCAACCAAT-3'
		R 5'-GTACACGGTGAGGAAGGTTTTA-3'
	E-selectin	F 5'-TGGAACACAACCTGTACATTTG-3'
		R 5'-AATTCCCAGATGAGGTACACTG-3'
Rat		
	Set8	5'-3'-GCAGGAAGAGAACTCCGTCG-3'
		5'-3'-AGAATCACATGACGGGGGTG-3'
	Foxo1	F 5'-GGTGAAGAGTGTGCCCTACT-3'
		R 5'-TCTTCTCCGGGGTGATTTCC-3'
	PTEN	F 5'-CCAGTCAGAGGCGCTATGTA-3'
		R 5'-TACATGAGCTTGTCCTCCCG-3'
	ICAM-1	F 5'-TCGGTGCTCAGGTATCCATC-3'
		R 5'-TGAGCTTCAGAGGCAGGAAA-3'
	E-selectin	F 5'-GCAAAGCTTCCCAGTGTGAA-3'
		R 5'-TTGAACACTGTACCCCTGCA-3'

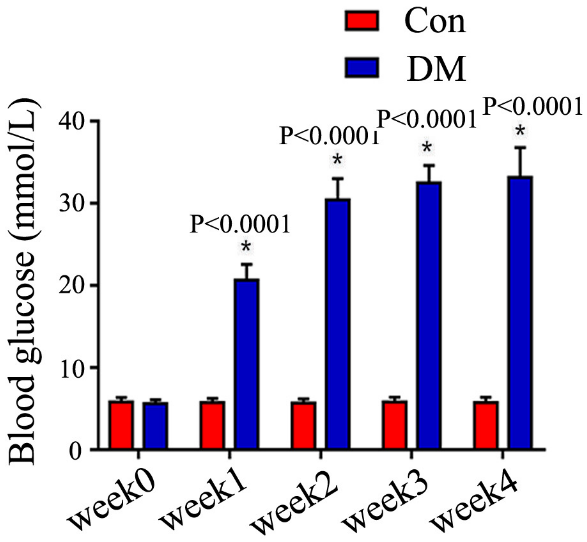

Con
DM
Blood glucose (mmol/L)
40
30
20
10
0
P<0.0001
*
P<0.0001
*
P<0.0001
*
P<0.0001
*
week0
week1
week2
week3
week4

Supplementary figure 1. Blood glucose levels in control and diabetic rats

Blood glucose levels across the control and diabetic groups started after the induction of diabetes in 4 weeks. (*$P<0.05$, compared with the control group, $n=5$/group)
